# An Inquiry, Critical and Experimental, into the Pathology of Fever

**Published:** 1853-12

**Authors:** N. S. Davis

**Affiliations:** Professor of Pathology, Practice of Medicine, and Clinical Medicine, in Rush Medical College and one of the Physicians to the Ill. Gen. Hospital


*An Inquiry, Critical and Experimental, into the Pathology of Fever.* By N. S. Davis M. D. Professor of Pathology, Practice of Medicine, and Clinical Medicine, in Rush Medical College and one of the Physicians to the Ill. Gen. Hospital.

[Continued from Page 464.]

On referring to the detail of those cases examined by Dr. Flint, and recorded in his third report, I find that in the *four* cases of *Typhus* examined, death took place on the 11th, 12th, 18th and 13th days respectively; while in the *four* cases of *Typhoid*, the deaths occurred on the 15th, 27th, 25th and 26th days after the commencement of disease. Thus showing a very marked difference between the *duration* of the cases of Typhus and Typhoid, and rendering it more probable that the intestinal lesions of the first were only an earlier stage of development of the same morbid process that was more advanced in the second. This view is further sustained by the well-known fact, that others have reported not only *slight* thickening of Peyer's glands in fatal cases of well marked Typhus, but complete *ulcerations*. Such was the case in the contagious epidemic of Typhus at Rheims, described by M. Landousky, where, in six autopsies, he found thickening and ulceration of Peyer's glands, and enlargement and softening of the mesenteric glands. Indeed, I think if M. Louis himself had found the same *slight* thickening or hypertrophy of the *eliptical plates* described by Dr. Flint in any of his cases of "*Simulated Typhoid affection*," he would have unhesitatingly marked it as genuine Typhoid fever. At the risk of being charged with presumption or temerity, I must express the opinion that there is a radical error in the mode of investigation adopted by M. Louis, and copied, with only slight modifications, by all his followers. This error consists in assuming it as a fact that there are two essentially different forms of continued fever, making the diagnosis during life in accordance with this assumption, rejecting altogether such cases as present an intermediate or doubtful grade of phenomena, and then adducing the differences in the symptoms and post-mortem appearances as proof of the correctness of the original division. I am very sure that the same mode of investigation, applied to other diseases, would compel us to acknowledge at least *three* specifically distinct forms of intermittent and remittent fe-

Reel: 78-10-5 END

ver, *two* of erysipelas, *two* of pneumonia, *three* of scarlatina, &c. Suppose we take, for instance, the well marked cases of pernicious or malignant intermittents and remittents, and, after the manner of Dr. Flint, carefully analyse the symptoms during life, and in fatal cases observe the post mortem appearances; and record both in one series of observed facts. Then take the equally well marked cases of *inflammatory* fevers of the same type, analyse their symptoms and post mortem phenomena, and record the same in another series of observed facts; taking care to exclude from both series all cases about which there could be any doubt as to their malignancy, on the one hand, or their inflammatory character, on the other. Does not every practitioner acquainted with these diseases, know that the two series thus made up, would present a far greater contrast in the symptoms during life, the post mortem appearances, the season and locality of prevalence, &c., than either Flint, Bartlett, or any other writer has presented between Typhoid and Typhus?

And yet no one doubts the fact that the cases in both series are mere varieties of the same disease, modified by climate, season, and local circumstances. And why do they not doubt their essential identity? Simply because they see in the same seasons and localities, besides the well marked cases of the two varieties named, numerous other cases presenting symptoms and morbid changes of every intermediate grade of character, until the two extremes are merged into each other so completely as to leave no definite line of distinction between them.

Take out all these intermediate cases, and you have left two diseases strongly contrasted in many essential particulars, relating both to functional and structural changes, with one prevailing most in warm climates, and the other in colder regions. But with the intermediate cases, they are at once inseparably linked together, and shown to be mere varieties of one and the same type of disease. Again, suppose we take scarlatina, and analyse all the cases of a well marked anginose variety in one series, and all those presenting only the phenomena of scarlatina simplex in another series; carefully leaving out of both series all cases so mixed in character as to make the practitioner doubt as to which series it properly belonged; is it not very apparent that the two series would present very marked differences in the relative extent and frequency of many of the leading symptoms, in the final results and in the post mortem appearances? But my readers are ready to exclaim that this is not a fair and legitimate mode of investigation. That to determine the true relations of any types, forms, or varieties of disease, we must take into the comparison *all* the cases that belong to those forms or varieties.

Thus, in ascertaining the relation between scarlatina simplex and scarlatina anginose, or between an inflammatory and pernicious intermittent, we must include all the intermediate cases, for the very important reason that *these* constitute the link or bond of union by which the other two are made to unite and show unmistakeably their affinity or relationship. But precisely here is the philosophical error of M. Louis and all his followers, in their investigations relative to *continued fevers.* M. Louis and his immediate co-laborers evidently commenced their pathological investigations in the proper mode, by including under one head all the cases of continued fever occurring in the Paris hospitals with which they were connected.

Finding, for a considerable length of time, a close similarity of symptoms during life, and pretty constantly, in fatal cases, evidences of some degree of disease of the follicles or glands of the small intestines, they at once proclaimed these intestinal lesions as the *essential* characteristic of the fever. But a continuance of the same investigations soon brought to light cases presenting the same phenomena during life, but without any visible intestinal lesions after death. These cases placed the investigator in a dilemma. To call them typhoid fever, would contradict the already published opinion that the lesions of the mucous membrane of the ileum were the *essential* characteristics of the fever. To call them by any other name would be to disregard the plainest identity of origin and symptoms. Hence there was no alternative but to call them "*simulated* typhoid fever," and place them in a separate list. Of course, after adopting this arrangement, M. Louis found more or less of the peculiar intestinal lesions present in *every* case of true *typhoid fever;* simply because all cases destitute of such lesions were placed in another list with another name, no matter what might have been their symptoms during life. So easy a method of disposing of a difficult question could not fail to find followers.

Hence, when Stokes and Graves, of Dublin; Watson, of London, and many others, found in the same hospitals and during the same seasons, half of the fatal cases of what they regarded as common continued fever, destitute of the lesions pointed out by Louis, they were at once told that they had confounded two essentially distinct diseases—that only those presenting the intestinal lesions were true typhoid, while all the rest were either *simulated* typhoid or typhus. Drs. Gerhard and Jackson, on their return from Paris, brought the same ideas to Philadelphia and Boston. Gaining access to the hospitals of those cities, they soon demonstrated by post mortem examinations, that the true typhoid intestinal lesions existed in a large proportion of the fatal cases of continued fever occurring in the wards, and straightway the Parisian distinction into typhoid and typhus was established on this side of the Atlantic. It is necessary to bear in mind that this distinction, both in Paris and elsewhere, had its origin exclusively in the morbid anatomy or post mortem appearances, without the least reference to the causes of the fevers or their symptoms during life. For, if two cases were brought from the same ship, or alley, or house, and one was found to linger with a continuous fever from three to six weeks, and terminate fatally, after more or less liquid discharges from the bowels and tympanitic distension of the abdomen, with visible thickening and ulceration of the glandular patches in the ileum, it was called true typhoid fever. But if the other betrayed a more profound depression of the vital powers, earlier delirium, a quicker pulse, more extensive engorgement of the spleen, lower lobes of the lungs and brain, and terminated fatally within the second or near the beginning of the third week, without any marked change in the glands of Peyer, it was, of course, typhus. Having thus founded a division of continued fevers on a particular anatomical lesion, the next step was to establish the diagnosis during life. To accomplish this the recorded histories of a long list of fatal cases, presenting the intestinal lesions, were carefully compared with an equal list of cases in which such lesions were absent. Such comparison, resulted in the discovery of certain general differences in the symptoms during life. For instance in the first list of cases diarrhœa or liquid stools were *more frequently* present than in the second, the abdomen was *more frequently* and decidedly tympanitic; when an eruption appeared over the chest or abdomen, it was in the form of *brighter red and more elevated spots*; the average duration of the disease was *longer*; and much the *larger* number of cases occurred during the summer and autumn months. These general differences, derived thus from *numerical averages*, were seriously arranged side by side in parallel columns, and handed down to us as the basis of diagnosis at the bed-side of the living patient. Guided by the general differences thus pointed out, most subsequent writers, especially in this country and on the continent of Europe, have endeavored to maintain the distinction. But to do so with some degree of clearness, they have uniformly been compelled to throw out of the comparison a considerable list of cases called *doubtful*. Not because it was doubtful whether they were genuine cases of continued fever, but because the symptoms belonging to the fever were so mixed as to render it *doubtful* whether they belonged to the list of typhoid or typhus. Thus Dr. A. Flint in his elaborate comparison of the histories of 164 cases of continued fevers, first avails himself of all the means of diagnosis pointed out by his predecessors, and then sets aside about one eighth of the whole number as *doubtful*. Not because he had any doubt about their being fair cases of continued fever, but because their symptoms were so mixed or of such a character as to leave some doubt whether in the aggregate they belonged to the typhoid or typhus class. Having set aside all the cases about which there could be any reasonable doubts, expressly as he says, to avoid including in either series of cases, any but such as were *well marked*, he finds on careful analysis and comparison sufficient differences to convince him of the essentially distinct characters of typhoid and typhus. The deceptive and illogical character of this mode of investigation will be made abundantly apparent, by applying it to the study of other diseases. Every practitioner extensively acquainted with remittent fever, as it presents itself through a series of years and in different localities, is aware that in so me seasons an localities a very large proportion of the cases, manifest an early tendency to the development of sub-acute inflammation of the portal viscera and intestinal mucous membranes, diminishing, in its progress, the *periodicity* of the febrile symptoms, and finally establishing a protracted typhoid condition, often ending in death; while in other seasons and localities, an equally large proportion of the cases manifest an early tendency to the development of cerebral, cerebro-spinal, and pulmonary congestions of sub-acute inflammations, and a much more speedy termination either in recovery or death.

It is equally well known that ordinary remittent fever seldom terminates fatally except by the supervention of some one or more of these local affections.

Now suppose some American Louis, located somewhere on the rich alluvial Prairies of the West, should chance to meet, for two or three years in succession, remittent fever manifesting chiefly the tendencies first named. If he makes a careful *post mortem* examination in each case he will find uniformly evidences of inflammation in the abdominal viscera, such as enlargement, induration, or suppuration of the Spleen or Liver or both, and redness, thickening, softening and perhaps ulceration of the mucous membranes. Evidences of these being uniformly present, though to a very variable extent, he confidently proclaims them as the essential and characteristic anatomical lesions of Remittent fever. But in the progress of time other cases even presenting the same general symptoms during their progress, and even terminate fatally and on examination none of the previously specified lesions are visible. These, however, are regarded as mere "*simulated*" Remittents. Meanwhile other practitioners occupying a field of practice in a different latitude habitually meet with many fatal cases of fever of a distinctly Remittent type, which in their character strictly correspond with the second class mentioned above.

On instituting careful post-mortem examinations the lesions are found not in the abdominal, but chiefly in the head and chest. Forthwith a controversy arises.

The Louis of the West has promulgated the doctrine that certain abdominal lesions are characteristic of Remittent fever, and many others on making examinations have found the same and corroborated his doctrine. But a large number of others, occupying different locations, have found in a majority of their fatal cases, such lesions entirely absent.

This leads to a careful comparison of the histories and symptoms during life, of those presenting *abdominal* lesions after death, with the histories of such as present anatomical lesions *only* in the head and chest. Taking the numerical averages derived from such comparison, and it is found that certain symptoms derived from disturbance of the cerebral and respiratory functions are earlier and more constantly present in the latter class of cases than in the former. They are also characterized by earlier prostration, and a shorter average duration of the disease. They, *too*, are found to occur more frequently in the spring and latter part of autumn. While the former class more frequently run a protracted course, present early indications of gastric, hepatic, and intestinal disturbances, a quicker and smaller pulse, more frequent tympanitic distension and tenderness of the abdomen, and occur much the most frequently during the months of July, August, and September. Finding the foregoing differences between the two classes of cases, to be pretty constant, Louis and his followers declare them to represent two essentially different fevers; the former class only, representing the true Remittent, and the latter a separate disease, which might be called periodical or malarious typhus. Still cases belonging to both classes are so frequently met with in the same locality, during the same season, and some of them presenting such a combination of the leading symptoms of both varieties, that a large proportion of practitioners continue to regard them as originating from the same general cause and as mere *varieties* of the same general type of fever. But some observer patiently records the the history of one or two hundred cases, placing all those presenting marked symptoms of abdominal complications in one list; those presenting no local complication or such only as occur in the head and chest in another; leaving all such as present symptoms so mixed as to render it difficult to say whether they ought to be ranked with one class or the other, to constitute a separate list of doubtfu cases.

Having thus obtained two lists of well marked cases, their comparison with each other, of course, discloses differences well calculated to corroborate the idea that they represent two essentially distinct fevers; and the observer becomes a convert to the doctrines of our supposed Louis. No one can fail to see the folly of such a mode of investigation. If you take a chain, the links of which are large at one end and taper gradually smaller as you approach the other, strike out a section in the middle embracing several links, and then compare the two ends, there will be no appearance of their ever having constituted parts of one continuous chain.

So if you take any general febrile disease, which is subject to great variations in its specific character, grade of severity, complications, and final tendencies, as it appears in different seasons, and contrast any two of its most strongly marked varieties, excluding all the intermediate links, they will most certainly present abundant differences both in the symptoms during life and in the anatomical lesions after death. But include in the comparison *all* the cases of the same type that occur during a series of years, and while you find one series of the cases prevailing most at one season, and presenting more prominently certain symptoms during life with corresponding lesions after death; and another series prevailing most at another season, with other symptoms most prominent followed by correspondingly different lesions; and you will also find numerous other cases not strictly belonging to either of these series, yet occurring indiscriminately with both, and presenting symptoms and lesions of such a character as to constitute a prefect bond of union between them, merging the two extremes so completely that "*no line of genuine distinction*" is left between them. And yet it is by unphilosophically setting aside these intermediate cases, thereby striking out the middle links of the chain, that a contrast is obtained sufficient to found a claim to specific difference.

To obviate the force of the foregoing illustration as applied to the different varieties of continued fever, some have suggested, that each variety constitutes a distinct and essential fever, arising from a distinct or specific cause; while those cases of intermediate grade, presenting mixed phenomena, have their origin from the co incident action of the causes of both inducing a blending of types and the production of a true hybrid disease, constituting the simulated typhoid of M. Louis and the doubtful cases of the other writers.

But this suggestion is more plausible than sound. For if the *causes* of both typhoid and typhus fevers are so closely related as to act simultaneously and conjointly upon the same individual, in such a manner as to produce an association of symptoms common to both, there must be a close analogy or mutual relationship between such causes. So much so, indeed, as to enable them to act *not only* on the same functions and vital properties, but also to influence such functions and properties in the same direction. This again, instead of sustaining the position that the varieties of continued fever, are so many essentially distinct diseases, would rather show that they were all variations of one general disease, such variations depending on diversities of season and peculiarities of cause, &c.

That we may have the causes of essentially different diseases acting upon the human system at the same time, and thereby inducing in the progress of the resulting disease, certain modifications of its symptoms I have no doubt. I have myself seen in a section of this city occupied chiefly by the *shanties* of poor foreigners, unmistakeable evidence of the co incident operation of malaria or the cause of Intermittents with all the circumstances usually regarded as efficient in developing typhus. In that locality I have seen the Intermittent cases presenting symptoms of unusual depression, such as a heavy depressed expression of the countenance, feebleness of the pulse, an unusual degree of languor during the intermission, and a greater proneness to dysentery; but aside from these modifications, the Intermittents and Remittents have uniformly presented their own peculiar and well known periodical phenomena from the beginning to the end. On the other hand, I have seen in the same district, late in the Autumn and during the Spring months, well marked cases of typhoid and typhus fevers, in which the only symptom of a *malarious* influence presented during their progress was a little greater tendency to daily remissions than usual; but after the continued fever had ceased and convalescence was fully established, the patients have been attacked with well marked paroxysms of Intermittent fever; requiring the usual anti-periodics to arrest their progress. In none of the cases, however, have I seen the *active* and *diagnostic* symptoms of the periodical and continued classes of fevers, combined so as to manifest themselves at the *same time* in the *same* patient. The two sets of causes may co-exist, but one or the other will predominate and give its own unmistakeable character to the disease until its force is spent, when the other may become predominant in its influence; as when ague is developed during the convalescence from continued fever. But I very much doubt whether two causes or agents essentially different in their properties, and capable of generating in the system, two distinct *general* diseases, can both operate simultaneously, in such a manner as to develope their own legitimate influences together; thereby constituting a true blending of distinct types or the production of a hybrid disease. They may so quickly follow each other in their effects, as to leave some of the more lasting symptoms of each visible at the same time. For instance, the specific poisons which cause the well known eruptive fevers, first affect the whole system, inducing general febrile action, and secondarily cause a local eruptive inflammation on the skin which may continue sometime after the general or constitutional action of the cause has ceased. Hence a patient may be exposed to the contagions of both Variola and Varicella, and after the first has induced its usual severe general fever and thrown out its specific cutaneous eruption, the latter may take effect and establish its peculiar eruption interspersed with the other. And so of other specific diseases. But we have no evidence that the constitutional or general febrile action of any two of them actually co-exists. There are, however, many diseases both general and local, which may be induced by the action of several causes. This is the case with nearly all the simple phlegmasia.

I think it is equally the case with the simple continued fever, or as Dr. G. B. Wood styles it, the "irritative fever." I have already defined *fever* to consist essentially, in a disturbance of the natural condition and relations of those properties which are inherent in all living organized matter. Hence it might be said with propriety that every cause or agent capable of acting on or disturbing such properties, throughout all the tissues, is sufficient to generate fever.

The particular type or fever will depend on the *specific direction* in which the vital properties are altered. Thus the contagious viruses that cause the acute Eruptive fevers, by their presence in the blood, simply *exalt* the universal and inherent susceptibility of the whole organism and soon establish a local disease in the cutaneous tissue. The exalted susceptibility and consequent universal functional disturbance, constitutes the *febrile stage*, which has no element or symptom that will uniformly or even generally serve to distinguish it from a simple ephemeral fever arising from any other cause. The specific modus operandi of these viruses is exhibited not in any uniform peculiarity of the febrile phenomena but in the accompanying or succeeding cutaneous affections. The former may arise from sudden exposure to cold, from over-exertion, from errors of diet, from teething and worms in children, from acute local inflammation in some important organ, and from any other cause capable of so increasing the organic susceptibility as to produce general functional disturbance. But in none of these cases will there be any specific cutaneous eruption Hence they are called by Dr. Wood irritative fevers; by Cullen they would be included in his class called Synocha; and by others they would be designated *simple continued fevers.*

Exaltation of the organic susceptibility, however, constitutes change in but one direction. Other causes may alter the same vital property in an opposite direction, or may continue this in the same direction and along with it develope other elements of morbid action so as to produce essentially different results. Hence instead of simple exaltation, there are many causes which directly lessen both the organic susceptibility and the vital affinity between the blood and the tissues. Such lead equally to universal functional disturbance, and consequently to general fever; but instead of irritative or inflammatory febrile action with increased sensibilities, they induce a grade of fever characterized by feebleness of organic action, blunted sensibility, and early disposition to prostration; constituting the *asthenic* or typhoid and typhus forms. Among the more common causes capable of altering the vital forces or properties in this direction, are the *protracted* influence of a low temperature; insufficient nourishment; foul air produced by too small, ill-ventilated, and over crowded apartments; and by the accumulation of filth and animal excretions in camps and jails, in emigrant ships, and the alleys and narrow streets of cities; and protracted mental anxiety and watchings, or depressing mental emotions.

All these, and others that might be enumerated, alter the elementary properties of the blood and tissues in the same *direction*, though not always to the same extent; and hence they all induce functional and febrile disturbances of the same general character. But there is still another cause or agent capable of inducing general fever, which cannot correctly be ranked with those that simply exalt or depress the organic properties. This agent is the specific cause of Intermittent and Remittent fevers. It is likened by Dr. Drake to the narcotico-irritant poisons, possessing a two-fold effect.

But the comparison is not strictly accurate. The malaria or specific cause of periodical fevers, exhibits no true *narcotic* properties; although its *irritant* qualities are plainly visible. By the latter it exalts in a marked degree the susceptibilities of the system giving to the febrile exacerbations a high grade of irritative excitement; while in the place of a *narcotic* influence, it produces such an effect on the vital affinities of the blood and tissues as to arrest the further formation of red corpuscles in the blood, and cause their usual relative proportion to diminish. In this, and the more distinctly paroxysmal character of the febrile exacerbations, consists the special peculiarities of what are styled malarious fevers. From all these observations it is evident that we must look for the *basis* of a rational *classification* of fevers, neither to the particular *causes*, nor to the *anatomical changes* revealed by post-mortem examinations; but to the specific *mode* or *direction* in which the *elementary properties* of the organism are altered in the development and active progress of the disease. On this basis we may reduce all the essential, idiopathic fevers to *three* great classes, viz.:

First: Those characterized by irritation or exaltation of the elementary properties, constituting irritative fever, and the co-existing development of a specific cutaneous or anginose inflammation. They are very properly styled the *Eruptive class*.

Second: Those characterized by irritation or exaltation of one elementary property, viz.: organic susceptibility, thereby developing true irritative fever ; by depression of tonicity or vital affinity in such a manner as to arrest the development of red corpuscles in the blood, inducing rapidly a change in the relative proportion of its constituents ; and by the whole duration of the disease being divided into well marked paroxysms or exacerbations and remissions. These are styled the *Periodical Class.*

Third : Those characterized by uniform diminution of the tonicity or vital affirmities of the organized textures, coupled with either simple exaltation or depression of the organic susceptibility, but without the exhibition of either uniform and successive paroxysms with impoverishment of blood globules, or specific and constant cutaneous eruptions. They may be called *Continued fevers.*

The first of these classes arises from specific contagions, each of which induces a pretty uniform series of morbid phenomena, among which is the cutaneous eruption which is of chief importance in determining the diagnosis. The prevalence and character of the fevers belonging to this class, are but little influenced by season, location or climate.

The second class arises also from one general specific cause ; and all the varieties tend to the production of the same class of morbid changes in the system, and to the same ultimate results. But the morbid phenomena resulting from the action of the specific cause, are sufficiently varied to require the division of the class into several distinct varieties or sub-divisions.

The varieties of fever composing the *third* class, have their origin in the action of a much greater number of causes, either acting together or singly, and hence they are much more varied in their active phenomena, average duration, and ultimate results.

None of them are *uniformly* characterized by specific eruptions, like the the first class, nor constant periodicity with rapid diminution of certain proximate elements of the blood, like those of the second; and yet cases frequently occur presenting scattered, non-inflammatory, but generally evanescent eruptions, sometimes in the form of elevated *rose colored spots*, and sometimes of a more papular appearance ; and other cases again present a visible tendency to exacerbations and remissions. But both the eruptions and the appearances of periodicity, occasionally presented by fevers of this class, are extremely variable, both in kind and degree; thereby presenting a marked contrast in these respects with the first two classes. Again, in proportion as the varieties of the third class arise from a greater number and combination of causes, they present a less constant and uniform succession of phenomena, and consequently admit of a less perfect and clearly defined diagnosis. The sub-divisions of the first class are based essentially on the specific characters of the eruptions belonging to each variety. Those of the second, are made to depend exclusively on the *degree* of *periodicity*. While those of the third, are based on the much less easily defined grade of febrile action, and the special direction given to the alteration of the elementary properties of the tissues; and hence they are much more likely to become insensibly merged into each other, leaving but an obscure line of of demarcation between them.

The following classification may be stated in the tabular form as follows, viz.:

| CLASSES. | VARIETIES. |
|---|---|
| 1st, Eruptive Fevers, | Variola,<br>Varicella,<br>Rubeola,<br>Roseola,<br>Scarlatina,<br>Erysipelas, |
| 2nd, Periodical Fevers, | Intermittent,<br>Remittent,<br>Yellow Fever. |
| 3d, Continued Fevers, | Simple Fever,<br>Complicated Fever,<br>Asthenic or Typhus Fever. |

A careful examination will show that each of the above classes, and all the varieties included in them, possess prominent and constant peculiarities, in reference both to their active phenomena and results, sufficient to entitle them to the grouping already named. Thus far I have said nothing concerning the Cullenian classificatin of fevers under the names Synocha, Synochus, and Typhus; not, however, because that arrangement was not founded on the actual phenomena of febrile affections, but because it was better fitted to characterize the different cases of the same class, and different stages of the same case. The essential foundation of the classification of Cullen, was the *grade* of febrile action. Thus his Synocha was a true inflammatory or stenic grade of fever; his Synochus commenced with a moderately stenic grade of action but soon verged into prostration; while his Typhus was from the beginning Asthenic. But every practitioner will find all these forms in each of the classes above name. Variola, for instance may present every *grade* of febrile action from the most inflammatory to the most asthenic or typhoid. So too with the periodical class. We find Intermittents and Remittents of an inflammatory or *Synocha* type; others beginning with this grade and running into a lower form, representing the *Synochus;* and still others even more asthenic than the most profound typhus. The space alloted to me in one number of the Journal will be devoted to an examination of the causes of fever, after which I will endeavor to point out the indications of treatment, indicated by the pathological views advocated in these articles.

